# Fractional order stagnation point flow of the hybrid nanofluid towards a stretching sheet

**DOI:** 10.1038/s41598-021-00004-3

**Published:** 2021-10-14

**Authors:** Anwar Saeed, Muhammad Bilal, Taza Gul, Poom Kumam, Amir Khan, Muhammad Sohail

**Affiliations:** 1 Faculty of Science, Center of Excellence in Theoretical and Computational Science (TaCS-CoE), King Mongkut’s University of Technology Thonburi (KMUTT), 126 Pracha Uthit Rd., Bang Mod, Thung Khru, Bangkok, 10140 Thailand; 2 Department of Mathematics, City University of Science and Information Technology, Peshawar, 25000 Pakistan; 3 Department of Medical Research, China Medical University Hospital, China Medical University, Taichung, 40402 Taiwan; 4 Department of Mathematics, Faculty of Science, King Mongkut’s University of Technology, Thonburi (KMUTT), 126 Pracha-Uthit Road, Bang Mod, Thrung Khru, Bangkok, 10140 Thailand; 5 Department of Applied Mathematics and Statistics, Institute of Space Technology, P.O. Box 2750, Islamabad, 44000 Pakistan

**Keywords:** Engineering, Mathematics and computing

## Abstract

Fractional calculus characterizes a function at those points, where classical calculus failed. In the current study, we explored the fractional behavior of the stagnation point flow of hybrid nano liquid consisting of TiO_2_ and Ag nanoparticles across a stretching sheet. Silver Ag and Titanium dioxide TiO_2_ nanocomposites are one of the most significant and fascinating nanocomposites perform an important role in nanobiotechnology, especially in nanomedicine and for cancer cell therapy since these metal nanoparticles are thought to improve photocatalytic operation. The fluid movement over a stretching layer is subjected to electric and magnetic fields. The problem has been formulated in the form of the system of PDEs, which are reduced to the system of fractional-order ODEs by implementing the fractional similarity framework. The obtained fractional order differential equations are further solved via fractional code FDE-12 based on Caputo derivative. It has been perceived that the drifting velocity generated by the electric field *E* significantly improves the velocity and heat transition rate of blood. The fractional model is more generalized and applicable than the classical one.

## Introduction

One of the relevant themes explored by researchers in the study of fluid properties on various mathematical models, includes multiple industrial and technological implications, such as elimination, wire drawing, glass fiber generation, assembly of elastic sheets, metallic plates cooling, and so on^1^. The non-Newtonian fluids streaming across the extended surface have attended great attention in recent decades. Non-Newtonian fluids' importance in a wide range of engineering and technical applications can never be overstated. Aerodynamics, plastic film emission, annealing, liquid film condensation phase, and copper wire thinning are only a few of the many applications^2^. Bhandari and Husain^3^ scrutinized the combined effects of rotational viscosity and magnetization force on 2D ferrohydrodynamic non-conducting nano liquid flow over a stretching surface while a stationary magnetic field was applied. Gul et al*.*^4^ demonstrated a computational model that was used to investigate the hybrid nanomaterials and enabled them to travel on a stretching sheet. The instability of fluid flow can be regulated using a magnetic dipole, according to their findings. Jawad et al*.*^5^ analyzed the convection flow of nanofluid across an extending surface comprising motile microorganisms, which resulted in the mutual transfer of heat and mass. The growing tendency in the magnetic field is thought to bring down the velocity and Nusselt number near the fluid's stretched surface. Srinivasulu et al*.*^6^ explored the role of an associated magnetic field on Williamson's ferrofluid on a stretch sheet using numerical methods. Khan et al*.*^7^ conducted a flow and thermal assessment for natural convection in a permeable trapezoidal cavity using a non-equilibrium thermal energy transition model. Paullet and Weidman^8^ have studied the stagnation point flow over an extending surface. Hamad & Ferdows^9^ further improved the idea about the stagnation point by adding porous media terminologies. Zainal et al*.*^10^ used the Matlab package bvp4c to analyze the time-dependent EMHD (electro-magnetohydrodynamic) stagnation point flow through *Al*_*2*_*O*_*3*_*-Cu*/water hybrid nano liquid involving a stretching and shrinking layer. Bejawada et al*.*^11^ reported the RK4 technique together with the shooting approach to study the MHD polar fluid across an infinite semistretched vertical porous substrate in the context of a heat source, magnetic field, and temperature.

For the last few decades, heat transfer through hybrid nanofluid has become a great research field of fluid mechanics. Heat transmission through hybrid nanofluids has a variety of industrial uses, including concrete heating and hot blended pavement in the concrete pavement sector, atomic reactors in the chemical industry, plastic factories for electric and mobile equipment and steam turbines, and glass fiber fabrication in the fiberglass industry^12^. Nanofluids are solid–liquid mixtures that have a carrier medium, such as base fluid, as well as nano-sized particles. Nanofluids have strong thermophysical properties due to their small dimensions (1–100 nm) and large specific surface area of nanomaterials; as a result, they can be used extensively in different areas of nanotechnologies^13^. Preparing hybrid (composite) nanoparticles can modify or alter the thermal conductivity of nanoparticles. Hybrid nanoparticles are nanoparticles made up of two or more different nanometer-sized materials. Hybrid nanofluids are fluids that have been made with hybrid nanoparticles. This analysis of hybrid nanofluids aims to improve heat transfer even further by increasing the thermal conductivity of these nanofluids. Researchers have used different sorts of hybrid nanoparticles in their studies. But here, keeping in view the applications and versatility of TiO_2_ and Ag nanoparticles, we have introduced TiO_2_ and Ag nanoparticles in human blood. Among the many metallic nanoparticles used in biomedical applications, silver nanoparticles (AgNPs) are one of the most significant and fascinating. Nanoparticles, particularly AgNPs, play an important role in nanobiotechnology, especially in nanomedicine. AgNPs have been focused on possible uses in cancer research and treatment, even though other noble metals have been used for different purposes^14^. Similarly, Titanium dioxide (TiO_2_) can be used in the shape of high-surface-area nanocrystals or nanodots. Magnetic properties can be found in them. Titanium oxide can also be known as Flamenco, titanium dioxide, rutile, and dioxo titanium. The ability of titanium oxide nanomaterials to suppress bacterial growth and prevent the development of new cell structures is well known^15^. That’s why its use more specious in human blood. Soomro et al*.*^16^ adopted the non-Newtonian Prandtl fluid framework to explore the role of thermophoresis and Brownian motion on MHD stagnation-point nanofluid flow along a lateral stretchable surface. Hamid et al*.*^17^ applied the Galerkin algorithm to numerically study the MHD flow of a nanofluid in diverging/converging channels. Chahregh et al*.*^18^ examined how a biological composite hybrid nano liquid made up of pure blood as the base fluid and TiO_2_ and Ag nanomaterials could be transported via an artery. The blood flow rather than closed channels are studied by the researchers in^19–21^ for testing purposes in medical laboratories. Liu et al*.*^22^ suggested a Pt/TiO_2_ nano-size particles for cancer cell therapy because noble metal particles are thought to improve TiO_2_ nanoparticles' photocatalytic operation. TiO_2_ and Au/TiO_2_ nanoparticles are also used to test the cancer slaying effect of our Pt/TiO_2_ nanocomposite.

The unreachable points where fundamental calculus fails can be characterized by fractional calculus. Fractional differential equations also referred to as exceptional differential equations, are a generalization of differential equations using fractional calculus^23^. Li et al*.*^24^ used Matlab fractional code Fde12 to develop a fractional model for Darcy hybrid nano liquid (Ag–MgO) flow over a porous swirling surface. They reported that silver Ag nano-size crystals' antibacterial properties could be used to control bacterial growth in a variety of applications, including dental practice, burns and wound care, surgery, and medicinal appliances. Mohammadein et al*.*^25^ proposed an approximate similarity approach for fluid flow across a vertical plate. To modify the partial differential equations into a resemblance ordinary differential equation with a fractional sense. Amin et al.^26^ have introduced the idea to transform the basic governing equations of fluid flow from PDEs into the fractional order ODEs. Mohammadein et al.^25^ have extended the above idea using the mathematical purely for the fluid flow analysis. Gul et al*.*^27^ analyzed fractional-order differential equations with momentum and thermal boundary layers and used the FDE-12 approach to transform the system of PDEs to a fractional-order system of ODEs. Using Caputo derivatives, Gul et al*.*^28^ investigated fractional-order 3D thin-film nanoliquid flow over an inclined accelerating surface. Hamid et al*.*^29,30^ introduced a combine approach toward Picard iterative and Chelyshkov polynomial method scheme. The suggested method's performance is evaluated using fractional order test problems and validated using numerical methods. Usman et al*.*^31^ developed a unique computational approach for computing stable solutions in multi-dimensions of time-fractional viscous Burger's models equation. Hamid et al*.*^32^ generalized the premise of classical Chelyshkov polynomials to functions with more than one variable, while providing evidence for theorems and definitions.

Keeping in view, the applicability of fractional derivative and the versatility of silver and titanium dioxide hybrid nanofluid using blood as base fluid, we have modeled the current problem. Which elaborate the fractional behavior of the 2D stagnation point flow of the hybrid nanofluid consisting of TiO_2_ and Ag nanoparticles across a stretching sheet. The fluid movement over a stretching layer is subjected to electric and magnetic fields. The problem has been formulated in the form of the system of PDEs, which are reduced to the system of fractional-order ODEs by means of new-similarity framework of the non-integer case. In the next section, the problem has been formulated, solved, and discussed.

## Mathematical formulation

Consider the two-dimensional stagnation point flow of the hybrid nanofluid consisting ${\text{TiO}}_{2} \& {\text{Ag}}$ nanoparticles towards a stretching sheet. The sheet is stretched with a stretching velocity $U_{w} = ax$. The blood is considered base fluid for testing purpose and medication. The electric and magnetic fields are jointly added to the model fluid flow over a stretching sheet. The solid nanoparticles and blood are supposed to be in thermal equilibrium including no-slip assumption in between the solid nanoparticles and base fluid. The basic equations are defined as:

1 $$ \frac{\partial u}{{\partial x}} + \frac{\partial v}{{\partial y}} = 0, $$

2 $$ \left( {u\frac{\partial u}{{\partial x}} + v\frac{\partial u}{{\partial y}}} \right) = u_{e} \frac{{du_{e} }}{dx} + \upsilon_{hnf} \frac{{\partial^{2} u}}{{\partial y^{2} }} + \frac{{\sigma_{hnf} }}{{\rho_{hnf} }}\left[ {E_{0} B_{0} - B_{0}^{2} \left( {u - u_{e} } \right)} \right], $$

3 $$ \left( {u\frac{\partial T}{{\partial x}} + v\frac{\partial T}{{\partial y}}} \right) = \frac{{k_{hnf} }}{{\left( {\rho Cp} \right)_{hnf} }}\frac{{\partial T^{2} }}{{\partial y^{2} }} + \frac{{\sigma_{hnf} }}{{\left( {\rho Cp} \right)_{hnf} }}\left[ {B_{0} \left( {u - u_{\infty } } \right) - E_{0} } \right]^{2} . $$

The physical conditions are.

4 $$ \begin{aligned} & u = u_{w} \left( x \right) = ax,v = 0,T = T_{w} ,\,\,at\,y = 0, \\ & u \to u_{e} = bx,\,\,\,\,T \to T_{\infty } ,\,\,\,\,\,\,\,\,\,\,\,\,\,at\,\,y \to \infty . \\ \end{aligned} $$

Here, $\rho ,\rho c_{p} ,\mu$ stand for the density, heat capacity and dynamic viscosity. Similarly, the velocity components are presented by $u{\text{ and }}v$.

The appropriate similarity variables are stated as^25–27^:

5 $$ u = axf^{\alpha } \left( \eta \right),v = - \sqrt {a\upsilon_{f} } f\left( \eta \right),\Theta \left( \eta \right) = \frac{{T - T_{\infty } }}{{T_{w} - T_{\infty } }},\eta^{\alpha } = \frac{{y^{\alpha } }}{\Gamma (\alpha + 1)}\sqrt {\frac{a}{{\upsilon_{f} }}} , $$

by using Eq. (5) in the Eqs. (1–4), Eqs. (2–4) are simplified as follows

6 $$ \begin{aligned} & \alpha^{2} \eta^{3\alpha - 3} f^{(\alpha + 2)} + \frac{{\rho_{hnf} }}{{\rho_{f} }}\frac{{\mu_{f} }}{{\mu_{hnf} }}\left[ \begin{gathered} \alpha^{2} (\alpha - 1)\left( {2\eta^{2\alpha - 3} + \eta^{2\alpha - 2} } \right)f^{\alpha + 1} + \alpha (\alpha - 1)^{2} \eta^{\alpha - 2} f^{\alpha } \hfill \\ + \alpha (\alpha - 1)\eta^{\alpha - 1} f^{\alpha } f + \alpha^{2} \eta^{2\alpha - 2} f^{\alpha + 1} f - \alpha^{2} \eta^{2\alpha - 2} \left( {f^{\alpha } } \right)^{2} + \lambda^{2} \hfill \\ \end{gathered} \right] \\ & \quad + \frac{{\mu_{f} }}{{\mu_{hnf} }}\frac{{\sigma_{hnf} }}{{\sigma_{f} }}\left( {\alpha \eta^{\alpha - 1} M} \right)\left( {E + \lambda - f^{\alpha } } \right) = 0, \\ \end{aligned} $$

7 $$ \begin{aligned} & \frac{{k_{hnf} }}{{k_{f} }}\left( {\alpha^{2} \eta^{2\alpha - 2} \Theta^{\alpha + 1} } \right) + \frac{{\left( {\rho Cp} \right)_{hnf} }}{{\left( {\rho Cp} \right)_{f} }}\left[ {(\alpha - 1)\eta^{\alpha - 2} \Theta^{\alpha } + \Pr \eta^{\alpha - 2} f\Theta^{\alpha } } \right] \\ & \quad + \frac{{\sigma_{hnf} }}{{\sigma_{f} }}\left( {\alpha \eta^{\alpha - 1} {\text{Ec}}M} \right)\left( {f^{\alpha } - (E + \lambda )} \right)^{2} = 0. \\ \end{aligned} $$

With inter-related conditions are:

8 $$ \begin{aligned} & f\left( 0 \right) = 0,f^{\alpha } \left( 0 \right) = 1,\Theta \left( 0 \right) = 1, \\ & f^{\prime}\left( \infty \right) = \lambda ,\Theta \left( \infty \right) = 0. \\ \end{aligned} $$

The above equations are modified as follows in the case of numeric derivatives:

9 $$ f^{\prime\prime\prime} + \frac{{\rho_{hnf} }}{{\rho_{f} }}\frac{{\mu_{f} }}{{\mu_{hnf} }}\left[ {ff^{\prime\prime} - \left( {f^{\prime}} \right)^{2} + \lambda^{2} } \right] + \frac{{\mu_{f} }}{{\mu_{hnf} }}\frac{{\sigma_{hnf} }}{{\sigma_{f} }}M\left( {E + \lambda - f^{\prime}} \right) = 0, $$

10 $$ \frac{{k_{hnf} }}{{k_{f} }}\Theta^{\prime\prime} + \frac{{\left( {\rho Cp} \right)_{hnf} }}{{\left( {\rho Cp} \right)_{f} }}\left[ {\Pr f\Theta^{\prime}} \right] + \frac{{\sigma_{hnf} }}{{\sigma_{f} }}EcM\left( {f^{\prime} - \left( {E + \lambda } \right)} \right)^{2} = 0. $$

Their boundary conditions are:

11 $$ \begin{aligned} & f\left( 0 \right) = 0,f^{\prime}\left( 0 \right) = 1,\Theta \left( 0 \right) = 1, \\ & f^{\prime}\left( \infty \right) = \lambda ,\Theta \left( \infty \right) = 0. \\ \end{aligned} $$

The magnetic field, Prandtl number, electric field parameter and Eckert number is expressed as:

12 $$ M = \frac{{\sigma B_{0}^{2} }}{{a\rho_{f} }},\Pr = \frac{{\nu_{f} }}{{\alpha_{f} }},E = \frac{{E_{0} }}{{B_{0} u_{w} }},{\text{Ec}} = \frac{{U_{w}^{2} }}{{Cp\left( {T_{w} - T_{\infty } } \right)}},\lambda = \frac{b}{a}. $$

### Mathematical models of thermophysical properties HNF

13 $$ \frac{{\mu_{hnf} }}{{\mu_{f} }} = \frac{1}{{(1 - \phi_{1} )^{2.5} \,\,\,(1 - \phi_{2} )^{2.5} }}, $$

14 $$ \frac{{\rho_{hnf} }}{{\rho_{f} }} = \left[ {\left( {1 - \phi_{2} } \right)\,\,\left\{ {1 - \left( {1 - \frac{{\rho_{{s_{1} }} }}{{\rho_{f} }}} \right)\,\,\phi_{1} } \right\} + \phi_{2} \frac{{\rho_{{s_{2} }} }}{{\rho_{f} }}} \right], $$

15 $$ \begin{aligned} \frac{{k_{hnf} }}{{k_{bf} }} & = \frac{{\left( {k_{{s_{2} }} + (m - 1)k_{bf} } \right) - (m - 1)\phi_{2} \left( {k_{bf} - k_{s2} } \right)}}{{\left( {k_{{s_{2} }} + (m - 1)k_{bf} } \right) + \phi_{2} \left( {k_{bf} - k_{s2} } \right)}}, \\ \frac{{k_{bf} }}{{k_{f} }} & = \frac{{\left( {k_{s1} + (m - 1)k_{bf} } \right) - (m - 1)\phi_{1} \left( {k_{bf} - k_{s1} } \right)}}{{\left( {k_{{s_{1} }} + (m - 1)k_{bf} } \right) + \phi_{1} \left( {k_{bf} - k_{s1} } \right)}}. \\ \end{aligned} $$

16 $$ \frac{{(\rho Cp)_{hnf} }}{{\left( {\rho Cp} \right)_{f} }} = \left[ {\left( {1 - \phi_{2} } \right)\left\{ {1 - \left( {1 - \frac{{\left( {\rho Cp} \right)_{{s_{1} }} }}{{\left( {\rho Cp} \right)_{f} }}} \right)\phi_{1} } \right\} + \phi_{2} \frac{{\left( {\rho Cp} \right)_{{s_{2} }} }}{{\left( {\rho Cp} \right)_{f} }}} \right], $$

17 $$ \frac{{\sigma_{hnf} }}{{\sigma_{bf} }} = \left[ {1 + \frac{{3\phi \left( {\phi_{1} \sigma_{1} + \phi_{2} \sigma_{2} - \sigma_{bf} (\phi_{1} + \phi_{2} )} \right)}}{{\left( {\phi_{1} \sigma_{1} + \phi_{2} \sigma_{2} + 2\phi \sigma_{bf} } \right) - \phi \sigma_{bf} \left( {\phi_{1} \sigma_{1} + \phi_{2} \sigma_{2} - \sigma_{bf} (\phi_{1} + \phi_{2} )} \right)}}} \right]. $$

### Physical quantities of interest

The expressions drag force $C_{fx}$ and Nusselt number ${\text{Nu}}_{x}$ are rebound as:

18 $$ C_{fx} = \frac{{\tau_{w} }}{{\frac{1}{2}\rho \left( {u_{w} } \right)^{2} }},\,\,\,\,\,\,\,{\text{Nu}}_{x} = \frac{{xq_{w} }}{{k\left( {T_{w} - T_{\infty } } \right)}}. $$

Applying Eqs. (5), transformations Eq. (10) yields

19 $$ C_{fx} {\text{Re}}_{x}^{0.5} = 2\alpha f^{\alpha + 1} \left( 0 \right),\,\,\,\,{\text{Nu}}_{x} {\text{Re}}_{x}^{ - 0.5} = - \Theta^{\alpha } \left( 0 \right). $$

## Caputo fractional derivatives

The fractional derivatives and related properties derived by Caputo are briefly discussed.

### **Definition 1**

Let $t > b,$ with the condition $b > 0\,,$ such that $b,\alpha ,t \in R$. The fractional derivative using the order $\alpha$ in $g \in C^{n}$ derived by Caputo is given by:

20 $$_{b}^{C} D_{t}^{\alpha } g\left( t \right) = \frac{1}{{\Gamma \left( {n - \alpha } \right)}}\int\limits_{b}^{t} {\frac{{g^{\left( n \right)} \left( \varsigma \right)}}{{\left( {t - \varsigma } \right)^{\alpha + 1 - n} }}} d\varsigma ,\,\,\,\,\,n - 1 < \alpha < n \in N. $$

### **Property 1**

*Let* $g\left( t \right),\,h\left( t \right):\left[ {a,\,b} \right] \to \Re$ *be such that* $_{b}^{C} D_{t}^{\alpha } g\left( t \right)$ *and* $_{b}^{C} D_{t}^{\alpha } h\left( t \right)$ *exist almost everywhere and let* $e_{1} ,\,e_{2} \in \Re .$ *Then* $_{b}^{C} D_{t}^{\alpha } \left\{ {e_{1\,} g\left( t \right) + e_{2\,} h\left( t \right)} \right\}$ *exists almost everywhere and*:

21 $$_{b}^{C} D_{t}^{\alpha } \left\{ {e_{1\,} g\left( t \right) + e_{2\,} h\left( t \right)} \right\} = e_{1} \,_{b}^{C} D_{t}^{\alpha } g\left( t \right) + e_{2} \,_{b}^{C} D_{t}^{\alpha } h\left( t \right). $$

### **Property 2**

*In this property, the function* $g\left( t \right) \equiv c$ *is considered constant and the fractional derivative of this function vanishes (approach to zero)*: $_{b}^{C} D_{t}^{\alpha } \,c = 0.$ *The Caputo idea used for the fractional-order differential equation as*:

$_{b}^{C} D_{t}^{\alpha } \,x\left( t \right) = g\left( {t,x\left( t \right)} \right),\,\,\,\,\,\alpha \in \left( {0,1} \right)$ *using the initial condition* $x_{0} = x\left( {t_{0} } \right).$

## Solution methodology

The transform Eqs. (6–7) and (8) are further altered as:

22 $$ \begin{aligned} & y_{1} = f,y_{2} = f^{\alpha } ,y_{3} = f^{\alpha + 1} ,y_{4} = \Theta ,y_{5} = \Theta^{\alpha } , \\ & y_{1} = 0, \, y_{2} - 1 = 0, \, y_{3} = 0,y_{4} - 1 = 0, \, y_{5} = 0. \\ \end{aligned} $$

23 $$ \left( \begin{gathered} \frac{{dy_{1} }}{d\eta } \hfill \\ \frac{{dy_{2} }}{d\eta } \hfill \\ \frac{{dy_{3} }}{d\eta } \hfill \\ \frac{{dy_{4} }}{d\eta } \hfill \\ \frac{{dy_{5} }}{d\eta } \hfill \\ \end{gathered} \right) = \left( {\begin{array}{*{20}l} {y_{2} } \hfill \\ {y_{3} } \hfill \\ { - \frac{{\rho_{hnf} }}{{\rho_{f} }}\frac{{\mu_{f} }}{{\mu_{hnf} }}\frac{1}{{\alpha^{2} \eta^{3\alpha - 3} }}\left[ \begin{gathered} \alpha^{2} (\alpha - 1)\left( {2\eta^{2\alpha - 3} + \eta^{2\alpha - 2} } \right)y_{3} + \alpha (\alpha - 1)^{2} \eta^{\alpha - 2} y_{2} \hfill \\ + \alpha (\alpha - 1)\eta^{\alpha - 1} y_{1} y_{2} + \alpha^{2} \eta^{2\alpha - 2} y_{1} y_{3} - \alpha^{2} \eta^{2\alpha - 2} \left( {y_{2} } \right)^{2} + \lambda^{2} \hfill \\ \end{gathered} \right]} \hfill \\ { + \frac{{\sigma_{hnf} }}{{\sigma_{f} }}\frac{{\mu_{f} }}{{\mu_{hnf} }}\frac{1}{{\alpha^{2} \eta^{3\alpha - 3} }}\left[ {\alpha \eta^{\alpha - 1} M} \right](E + \lambda - y_{2} )} \hfill \\ {y_{5} } \hfill \\ { - \frac{{\left( {\rho Cp} \right)_{hnf} }}{{\left( {\rho Cp} \right)_{f} }}\frac{{k_{f} }}{{k_{hnf} }}\frac{1}{{\alpha^{2} \eta^{2\alpha - 2} }}\left[ {(\alpha - 1)\eta^{\alpha - 2} \Theta^{\alpha } + \Pr \eta^{\alpha - 2} f\Theta^{\alpha } } \right]y_{5} } \hfill \\ { - \frac{{\sigma_{hnf} }}{{\sigma_{f} }}\frac{{k_{f} }}{{k_{hnf} }}\frac{1}{{\alpha^{2} \eta^{2\alpha - 2} }}\left[ {\alpha \eta^{\alpha - 1} MEc} \right]\left( {y_{2} - (E + \lambda )} \right)^{2} ,} \hfill \\ \end{array} } \right), $$

## Results and discussion

**Figure 1:**
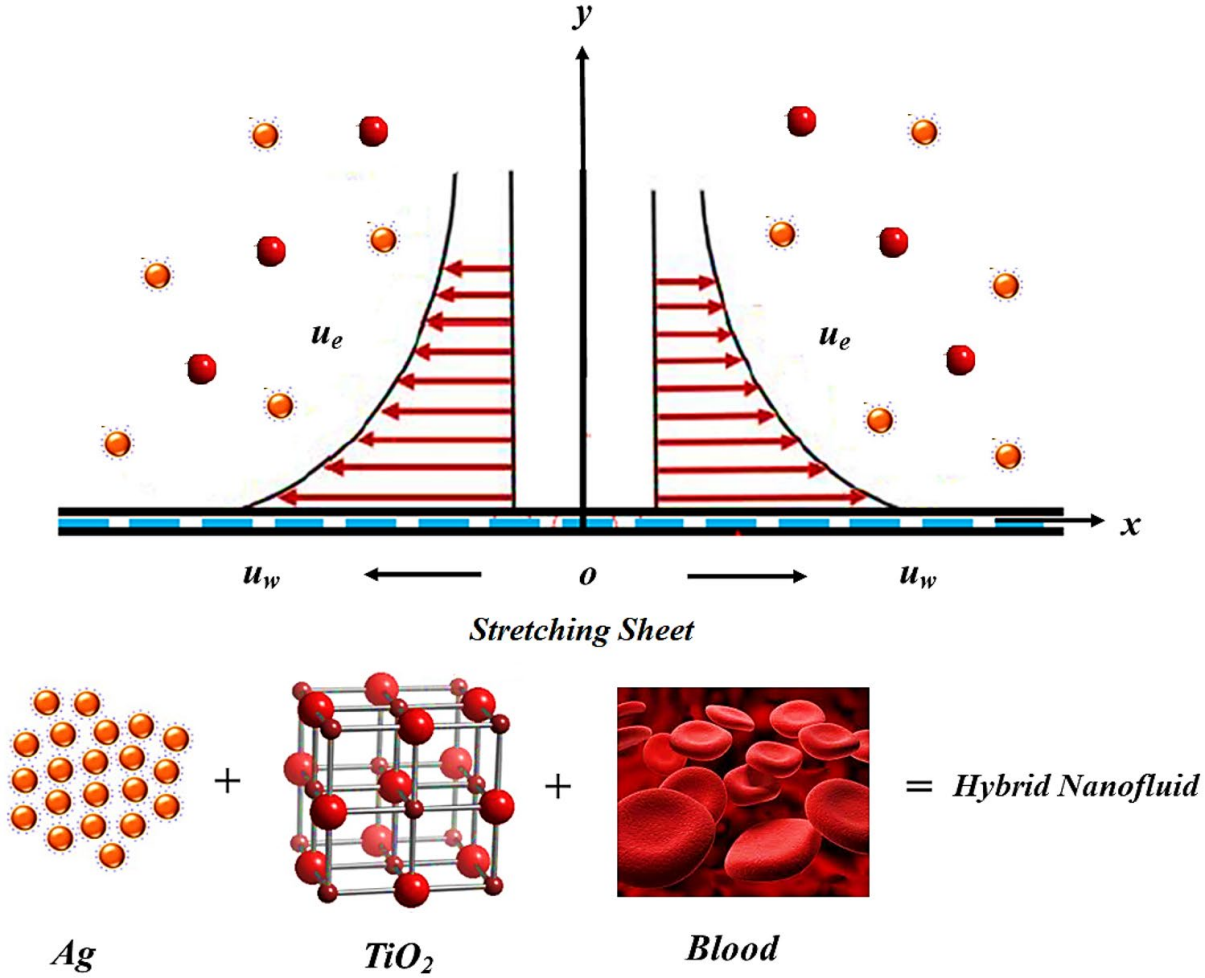
Physical Presentation of the problem.

**Figure 2:**
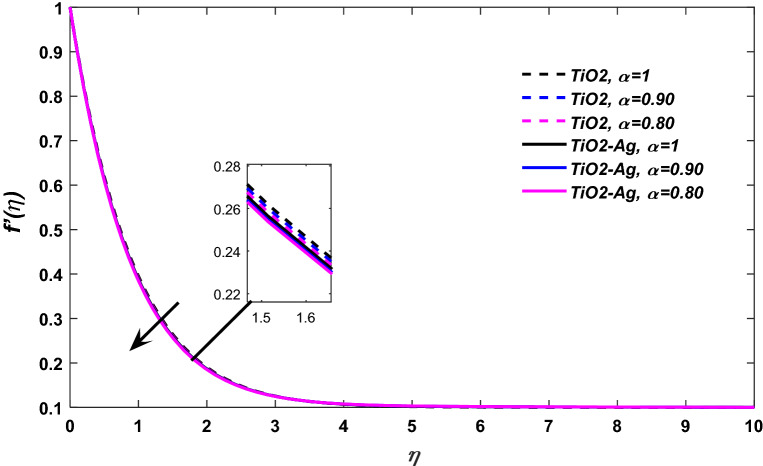
$\alpha$ versus velocity field, When $E = \lambda = 0.1,\phi_{1} = \phi_{2} = 0.02,M = 0.1,\Pr = 21.$

The stagnation point flow of the hybrid nanofluid considering blood as a base fluid towards a stretching surface is analyzed in this research. The solid line in the figures shows the hybrid nanofluid consisting of $({\text{TiO}}_{2} \& {\text{Ag}})$ while the dotted line shows $({\text{TiO}}_{2} )$ nanomaterials. Figure 1 illustrates the blood mechanism over a stretching sheet. Figure 2 revealed the consequences of variation in fractional order $\alpha$ versus velocity profile $f^{\alpha }$. It has been perceived that the fluid has a maximum velocity at $\alpha = 1,$ but gradually the fluid velocity start declination with decreasing values of $\alpha .$

**Figure 3:**
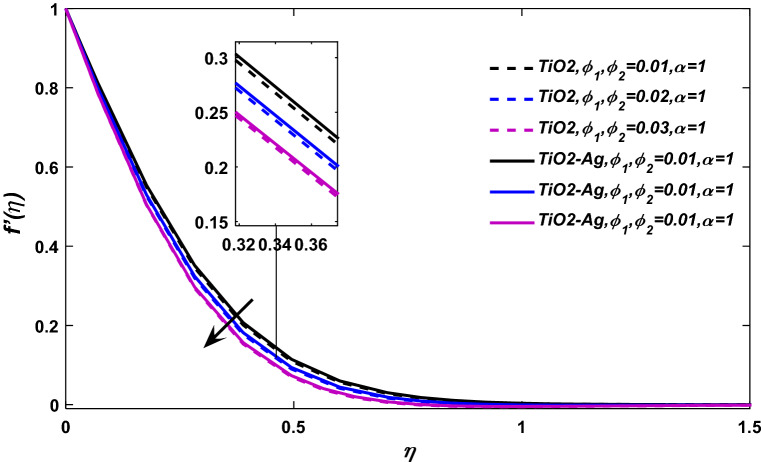
$$\phi_{1} ,\phi_{2}$$ versus $$\frac{df}{{d\eta }}$$, when $$\lambda = 0.01,E = 0.1,\alpha = 1,M = 0.1,\Pr = 21.$$

**Figure 4:**
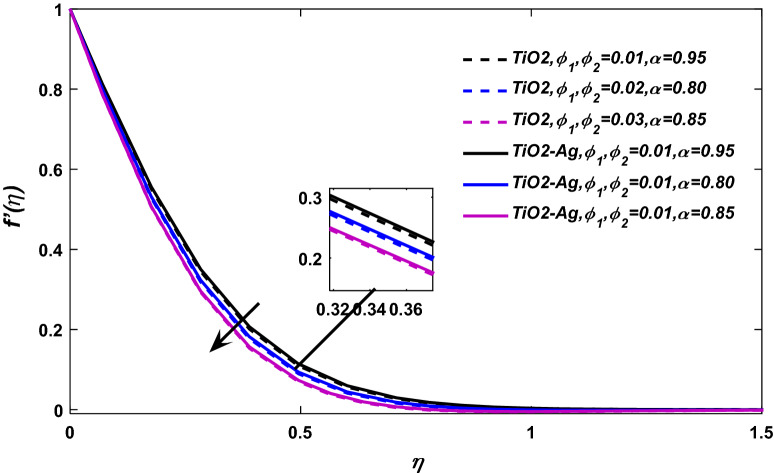
$\phi_{1} ,\phi_{2}$ versus velocity field when $\alpha = 0.95,0.90.0.85,\lambda = 0.01,E = M = 0.1,\Pr = 21.$

Figures 3 and 4 display the behavior of velocity field against volume friction parameters $\phi_{1} \,= \,{\text{TiO}}_{2}\, \,{\text{and}}\,\,\phi_{2} \,=\, {\text{Ag}}$ respectively. The blood flow shows a positive response versus a rising amount of Titanium dioxide $({\text{TiO}}_{2} )$ and silver $({\text{Ag}})$ nanocomposites. Because, the specific heat capacity of blood is greater than titanium dioxide and silver, so the increasing number of these nanoparticles reduces the average heat capacity of the blood, eventually blood losses its viscosity, and as a result, the fluid velocity enhances.

**Figure 5:**
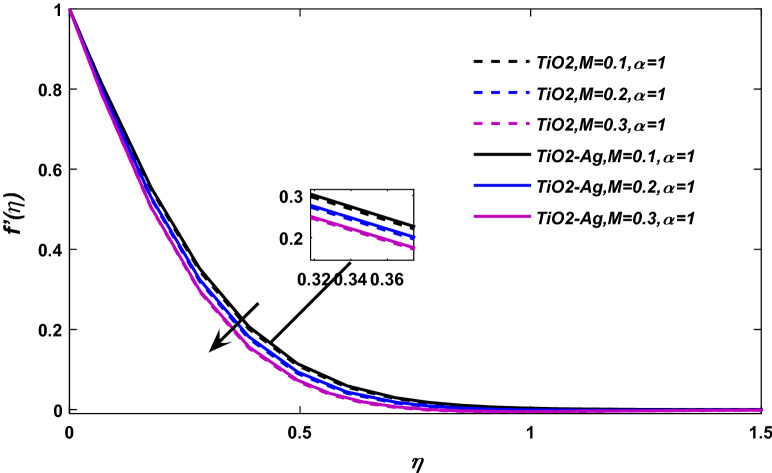
$M$ versus velocity field when $E = 0.1,\lambda = 0.01,\phi_{1} ,\phi_{2} = 0.02,\alpha = 1,\Pr = 21.$

**Figure 6:**
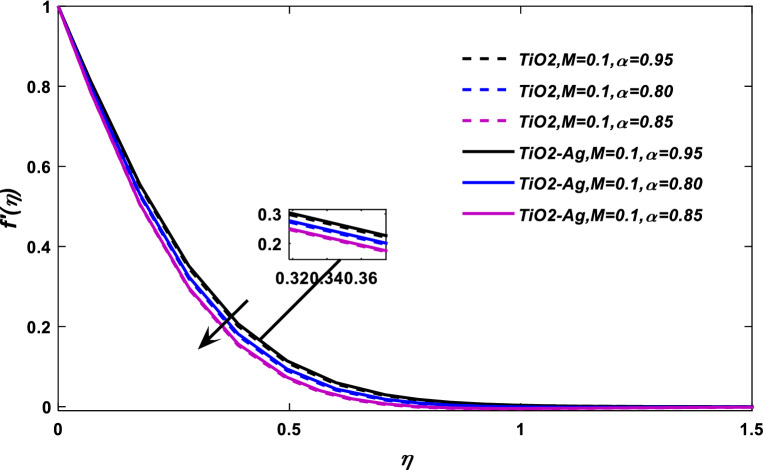
$M$ versus velocity field when $E = 0.1,\lambda = 0.01,\phi_{1} ,\phi_{2} = 0.02,\Pr = 21.$

The consequences of magnetic force parameter *M* on velocity field have been illustrated through Figs. 5 and 6, respectively. Figure 5 shows the classical case at $\alpha = 1,$ and Fig. 6 at the non-integer case $\alpha = 0.95,\,0.90,\,0.85.$ The magnetic effects generate Lorentz force (The Lorentz Force is the effect exerted by electric and magnetic fields on an electric charge particle), which retard the flow field, as a result, the fluid velocity reduces.

**Figure 7:**
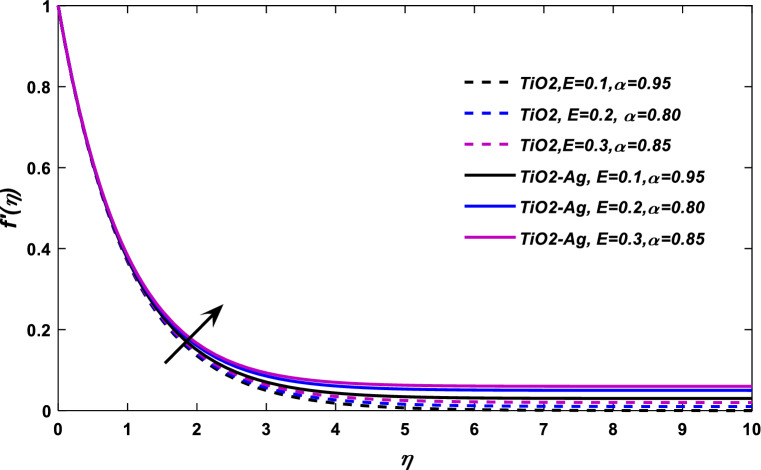
$E$ versus velocity field when $M = 0.1,\lambda = 0.05,\phi_{1} ,\phi_{2} = 0.02,\Pr = 21.$

**Figure 8:**
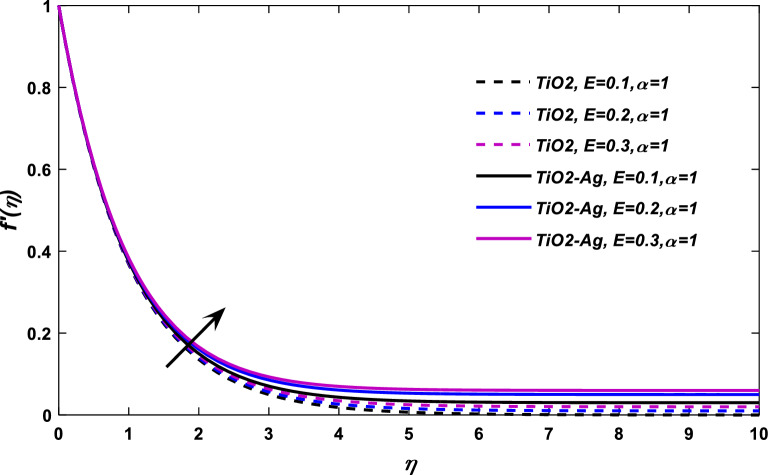
$E$ versus velocity field when $M = \lambda = 0.1,\phi_{1} ,\phi_{2} = 0.02,\Pr = 21.$

Figures 7 and 8 reported the nature of fluid velocity against electric force parameter $E$ at classical order $\alpha = 1,$ and fractional case, respectively. The velocity profile $f^{\alpha }$ show a positive response with the action of electric force effect *E*. When an electric effect is applied on the flow field, the free electron arranges the flow field in the form of drift velocity. The relative speed of a particle, such as an electron, subjected to an electric field is referred to as drift velocity. The free electrons form an electric field added to the blood flow and on behalf of drift velocity, these electrons compel the blood to flow in a specific direction, which eventually improves its velocity.

**Figure 9:**
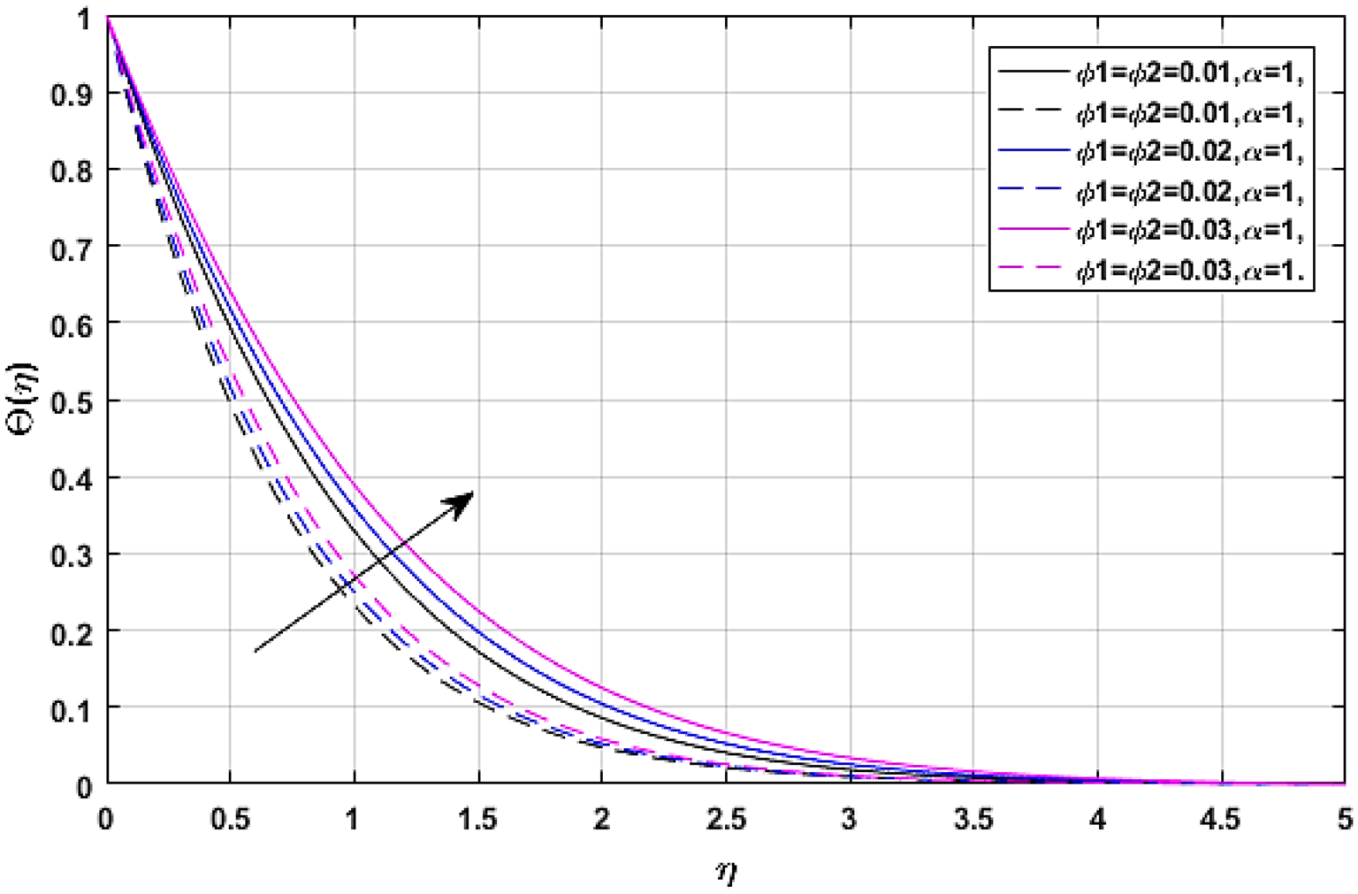
$\phi_{1} ,\phi_{2}$ versus temperature field when $M = \lambda = E = {\text{Ec}} = 0.1,\Pr = 21.$

**Figure 10:**
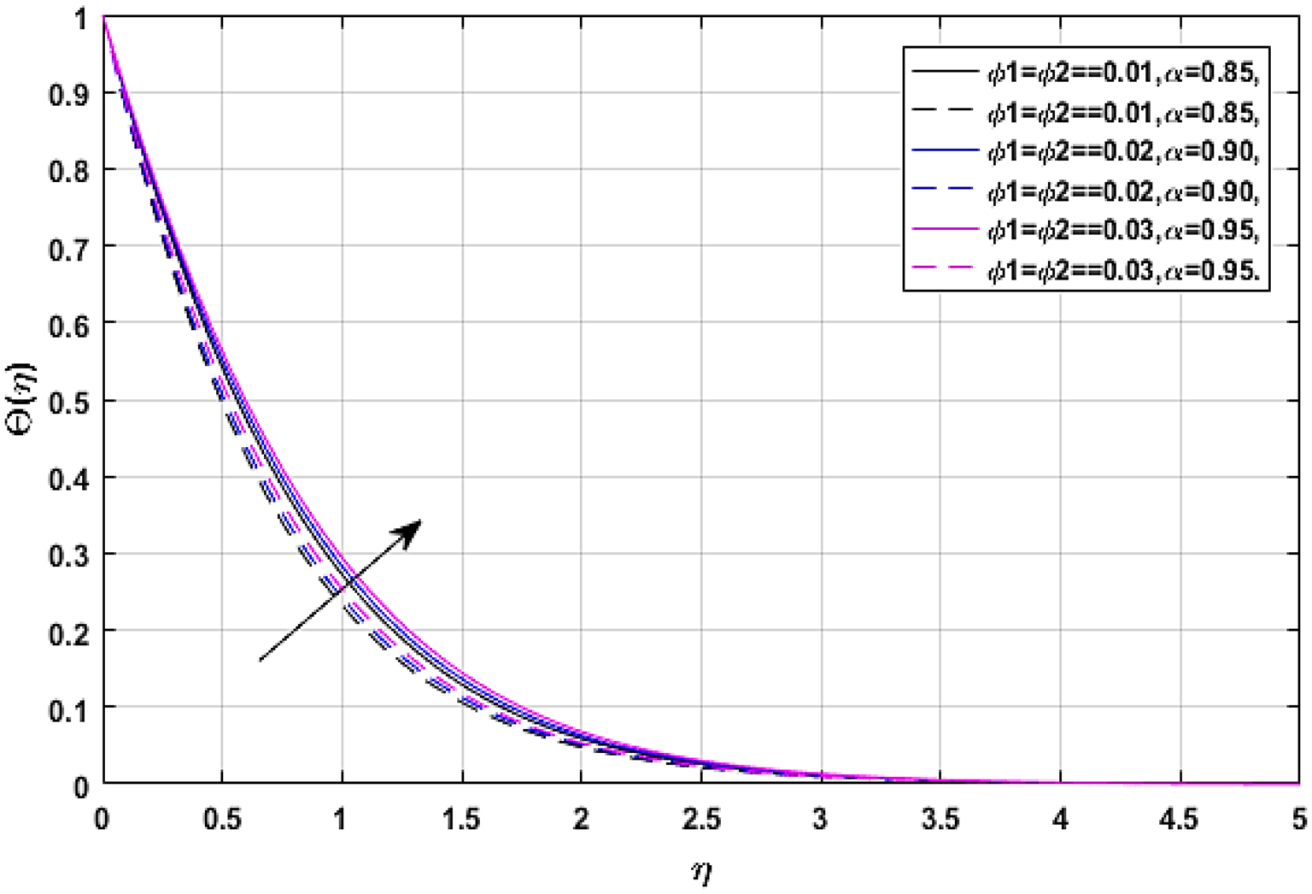
$\phi_{1} ,\phi_{2}$ versus temperature field when $M = \lambda = E = {\text{Ec}} = 0.1,\Pr = 21.$

Figures 9 and 10 present the relation of volume fraction parameters $\left( {\phi_{1} = {\text{TiO}}_{2} \,{\text{and}}\,\,\phi_{2} = {\text{Ag}}} \right)$ and thermal energy profile $\Theta \left( \eta \right).$ Figure 9 revealed the classical case at $\alpha = 1,$ and Fig. 10 report the non-integer case, respectively. The fluid temperature $\Theta \left( \eta \right)$ rises with the effects of volume fraction parameters. Because as we have discussed earlier that the specific heat capacity of blood is greater than titanium dioxide and silver, so the increasing number of these nanoparticles reduces the average heat capacity of the blood, eventually blood losses its viscosity due to excessive heat and cause the enhancement of fluid temperature.

**Figure 11:**
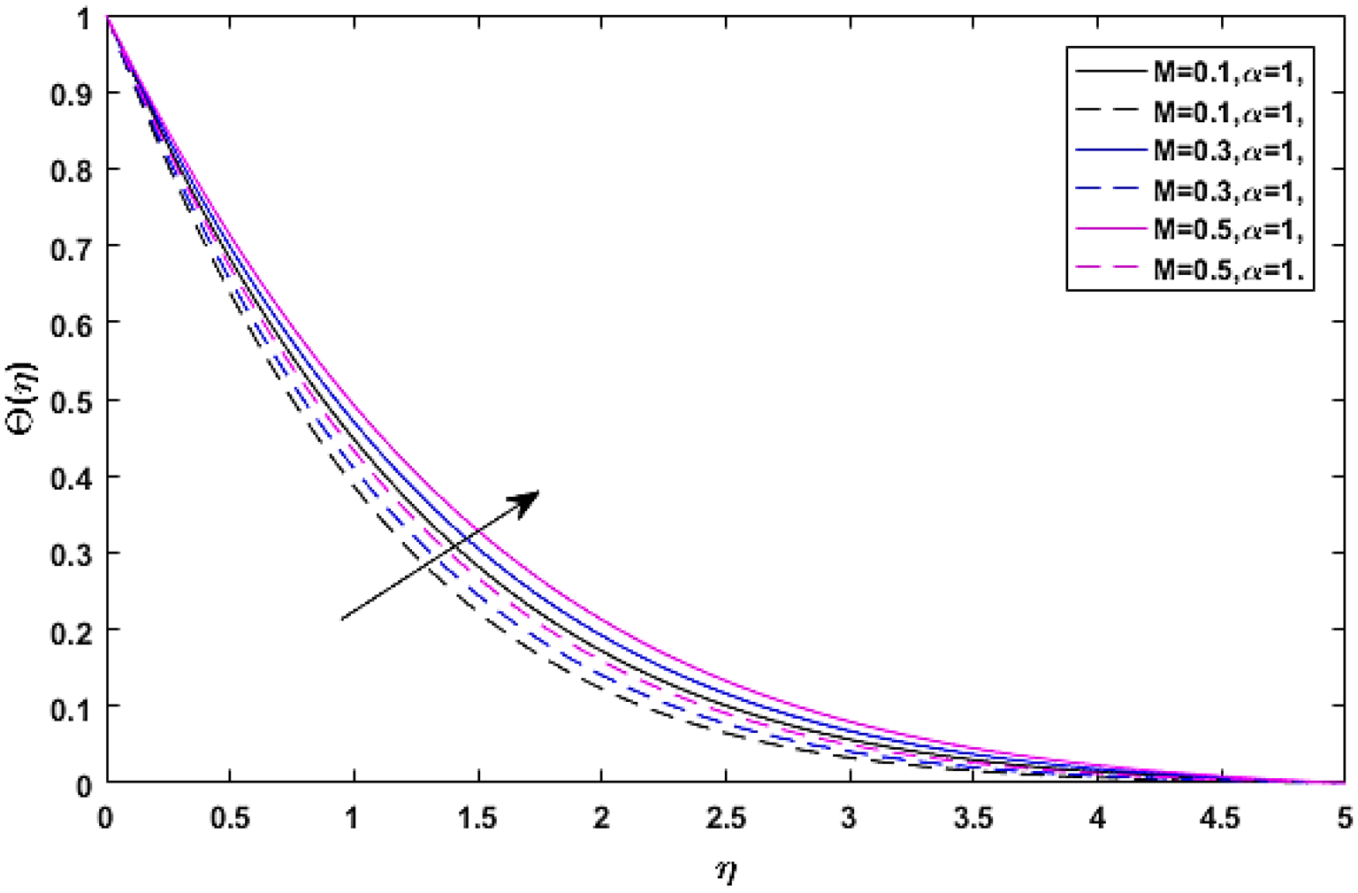
$M$ versus temperature field when $\lambda = E = {\text{Ec}} = 0.1,\phi_{1} = \phi_{2} = 0.02,\Pr = 21.$

**Figure 12:**
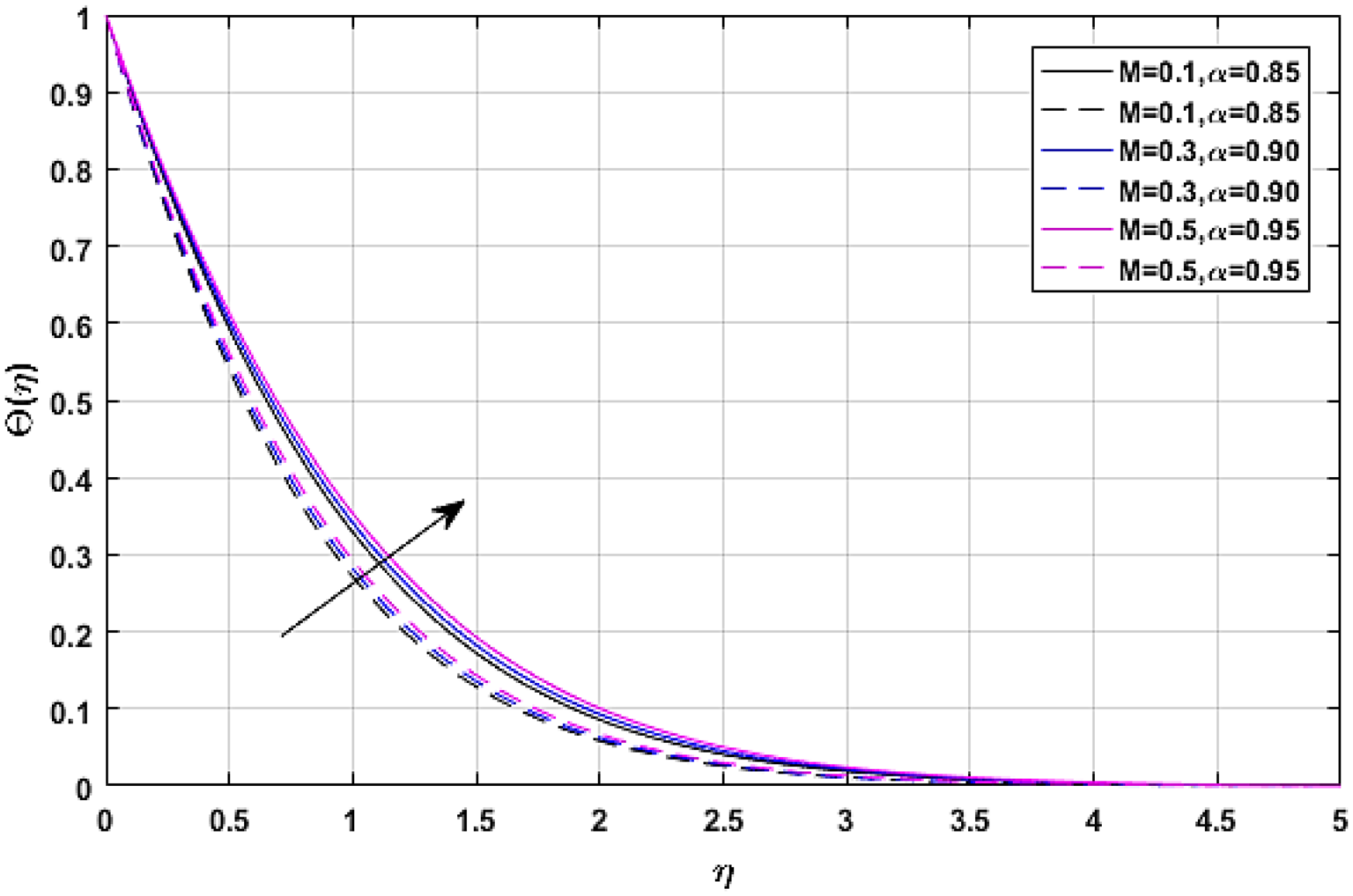
$M$ versus temperature field when $\lambda = E = {\text{Ec}} = 0.1,\phi_{1} = \phi_{2} = 0.02,\Pr = 21.$

The consequences of magnetic force coefficient *M* on temperature profile $\Theta \left( \eta \right)$ is elaborated through Figs. 11 and 12 respectively. The opposing force which is generated due to the magnetic effect causes friction between the flow field and free ions. That friction force produces some amount of heat, which when added to the fluid, raises the average temperature of the fluid.

**Figure 13:**
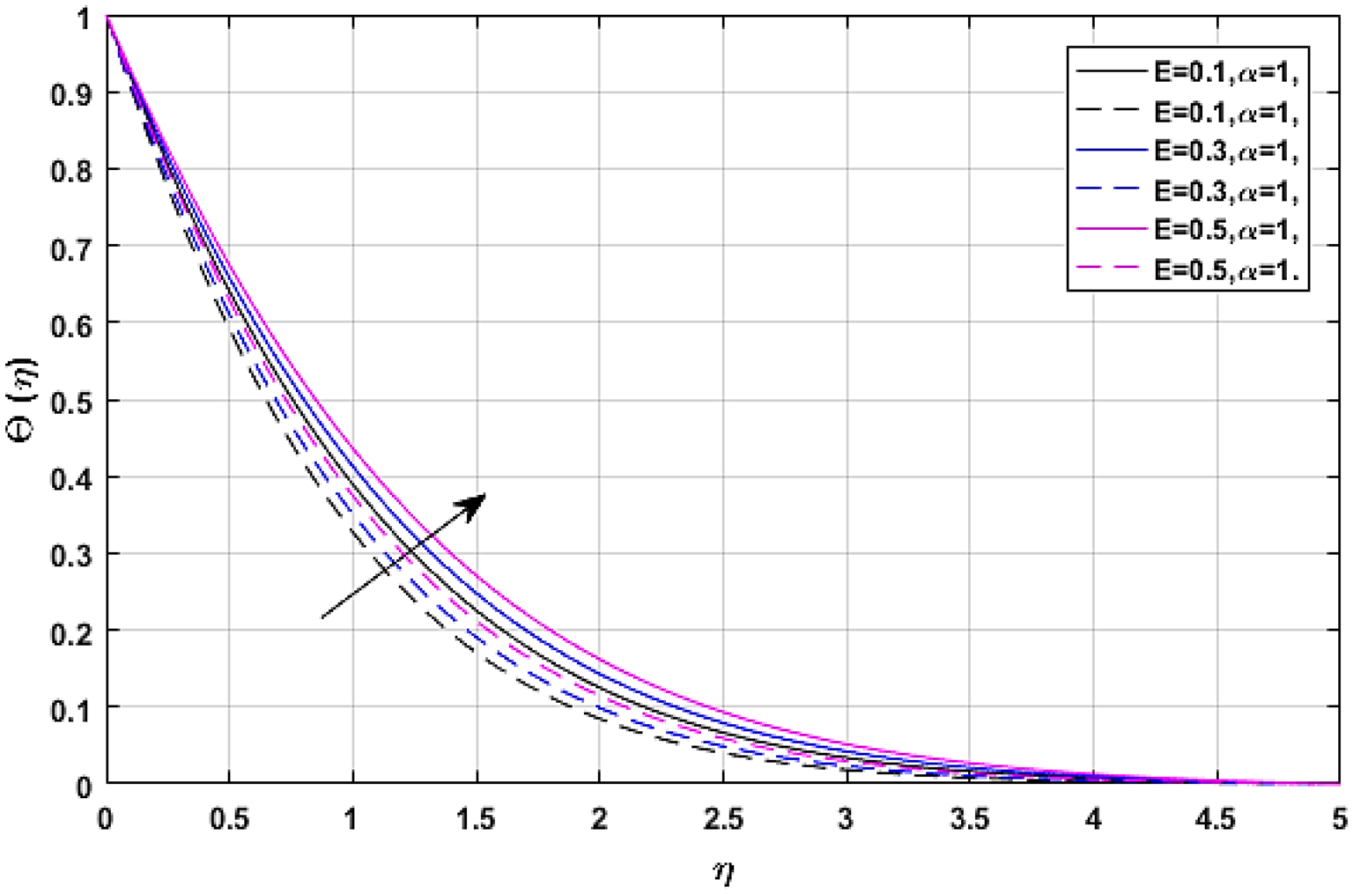
$E$ versus temperature field when $\lambda = M = {\text{Ec}} = 0.1,\phi_{1} = \phi_{2} = 0.02,\Pr = 21.$

**Figure 14:**
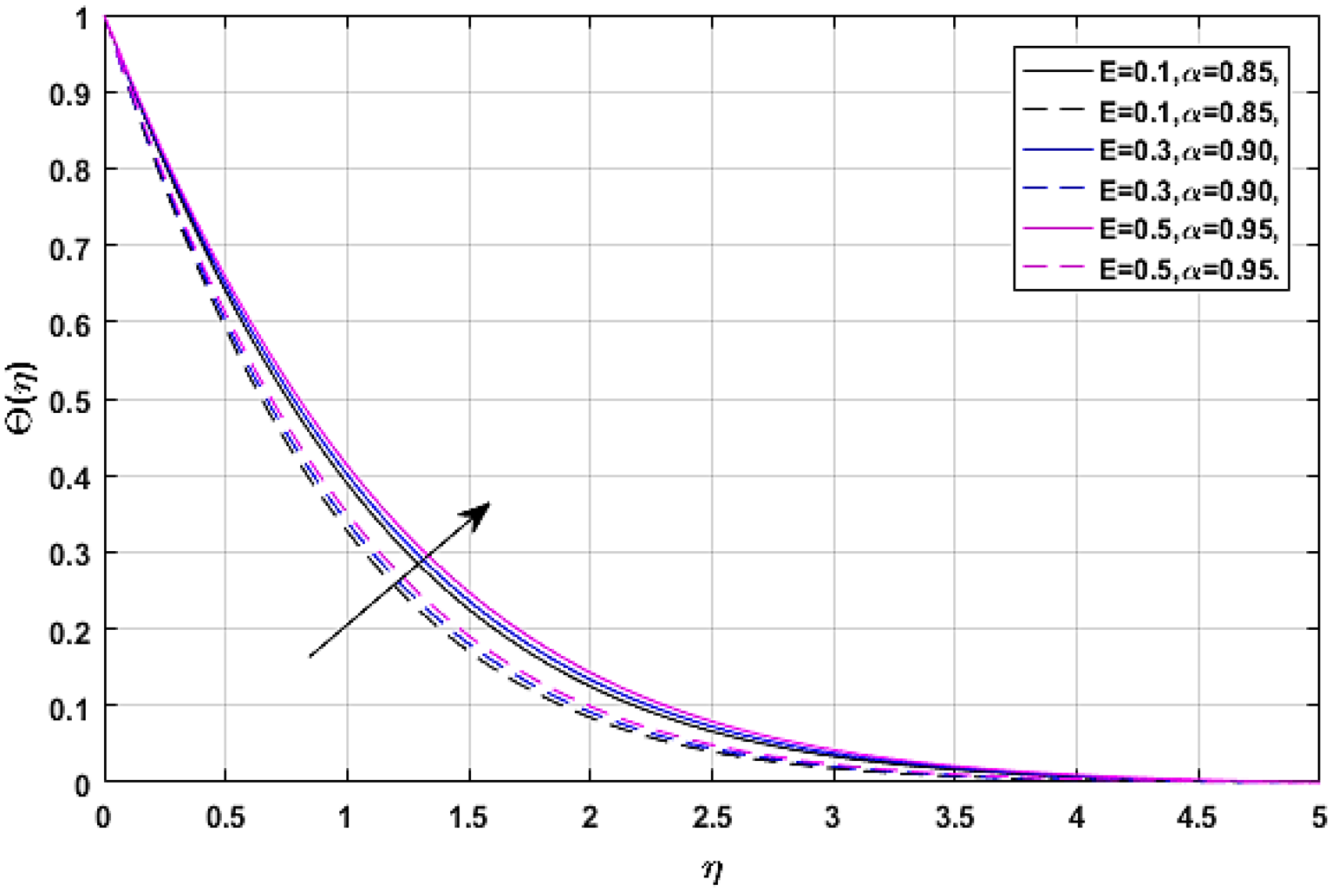
$E$ versus temperature field when $\lambda = M = {\text{Ec}} = 0.1,\phi_{1} = \phi_{2} = 0.02,\Pr = 21.$

**Figure 15:**
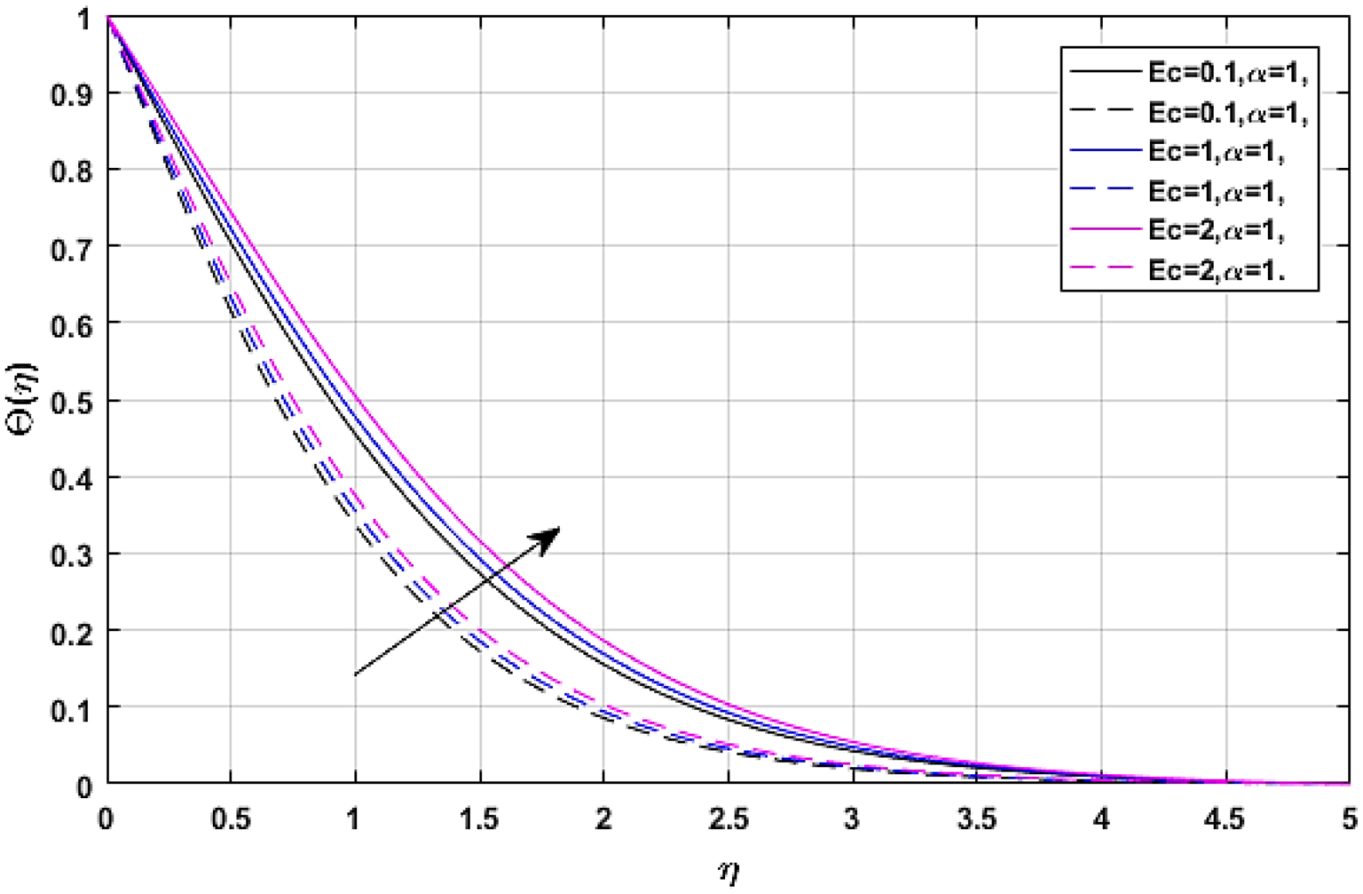
${\text{Ec}}$ versus temperature field when $\lambda = M = E = 0.1,\phi_{1} = \phi_{2} = 0.02,\Pr = 21.$

**Figure 16:**
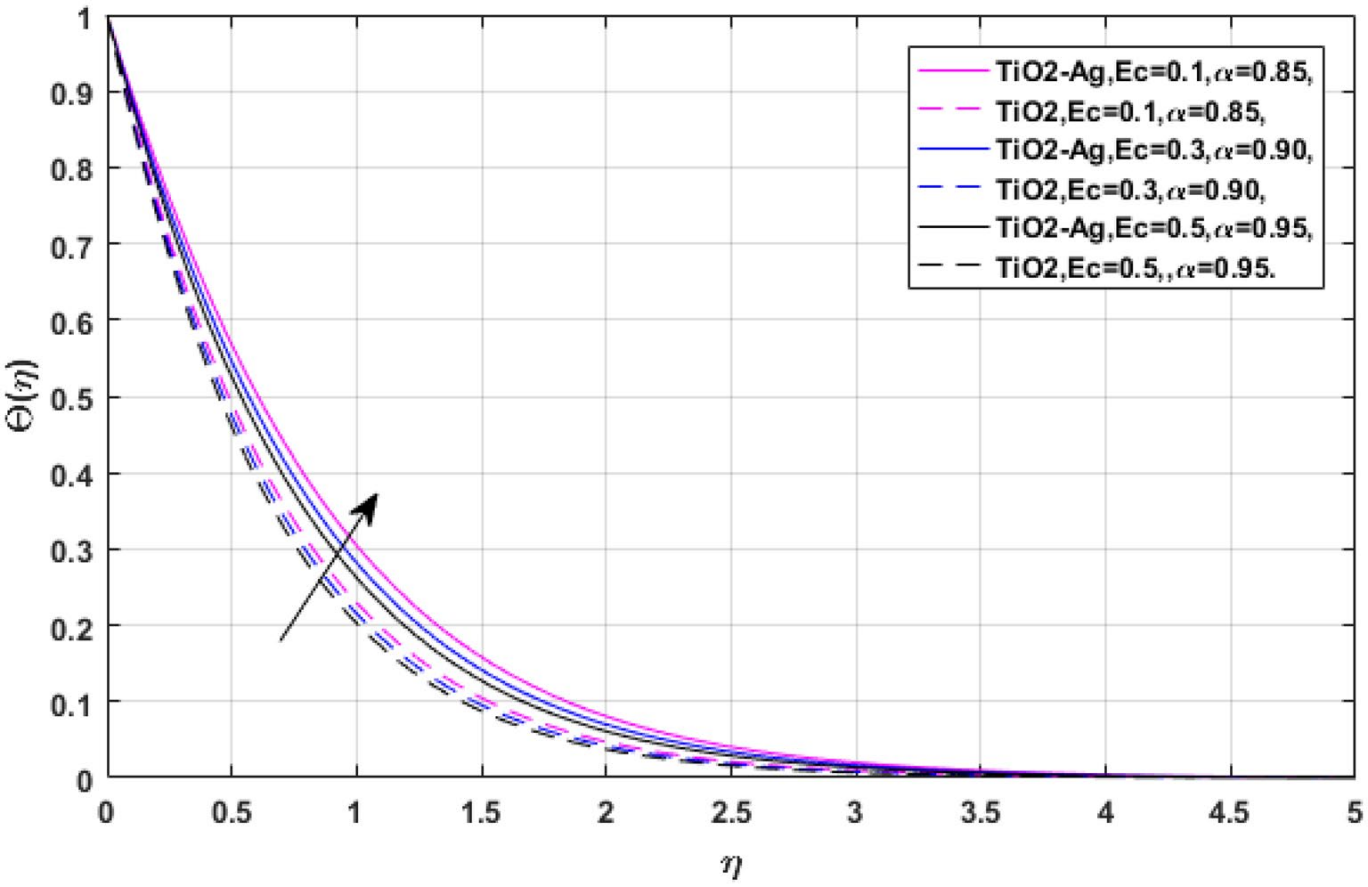
${\text{Ec}}$ versus temperature field when $\lambda = M = E = 0.1,\phi_{1} = \phi_{2} = 0.02,\Pr = 21.$

Figures 13 and 14 manifest the nature of thermal energy $\Theta \left( \eta \right)$ transition versus electric effect parameter *E*. The temperature profile enhances with the increment of electric force factor *E*. because the rising trend in electric effect boosts the kinetic energy between fluid molecules, which encourages fluid temperature to improve. The Eckert number upshot on the energy profile has been displayed via Figs. 15 and 16. In which, Fig. 15 revealed the classical case at $\alpha = 1,$ and Fig. 16 report the non-integer case, respectively. The Eckert number describes the relationship between enthalpy and kinetic energy in a flow. It involves the activities performed against viscous fluid pressures to convert kinetic energy into internal energy. A rise in the Eckert number indicates that the kinetic energy of the fluid is high, resulting in increased fluid vibration and increased fluid-molecule collisions. Increased molecule collisions increase heat dissipation in the boundary layer field, increasing the temperature profile.

Table 1 analyzed the drag force and heat transfer for the fractional and integer orders. It can be perceived that $\alpha = 1,$ the velocity and heat transfer rate show their maximum, while in non-integer case the fluid temperature and velocity both reduce. Tables 2 and 3 express the behavior of skin friction and Nusselt number versus embedding parameters, respectively. The rising credit of magnetic *M* and electric *E* parameters both enhances the skin friction as shown in Table 2. On the other hand, the increasing values of Eckert number Ec and volume frictions parameters $\left( {\phi_{1} \,{\text{and}}\,\,\phi_{2} } \right)$ significantly enhance the heat transmission rate illustrated via Table 3. The comparison of the present work with published considering integer order study has been displayed in Table. 4. The common parameters are chosen and closed agreement achieved.

**Table 1:** The fraction and integer order representation of the drag force and heat transfer coefficient.

$\alpha$	$$\begin{aligned} & f^{\alpha + 1} (0) \\ & {\text{TiO}}_{2} + {\text{Ag}} \\ \end{aligned}$$	$$\begin{aligned} & - \Theta^{\alpha } (0) \\ & {\text{TiO}}_{2} + {\text{Ag}} \\ \end{aligned}$$	$$\begin{aligned} & f^{\alpha + 1} (0) \\ & {\text{TiO}}_{2} \\ \end{aligned}$$	$$\begin{aligned} & - \Theta^{\alpha } (0) \\ & {\text{TiO}}_{2} \\ \end{aligned}$$
1	0.36930170609170376	0.6438542481228724	0.3513618613256846	0.6395896647232362
0.95	0.3476249888802566	0.6016610097711959	0.3314005479483403	0.600484009045378
0.90	0.32405829999296015	0.49831893466281774	0.3094423537682283	0.4045916664571034
0.85	0.2990170562515085	0.21099976910025595	0.285929688052459	0.2005682563030754

When $$M = {\text{Ec}} = {\text{Ec}} = \lambda = 0.1,\phi_{1} = \phi_{2} = 0.02,\Pr = 21.$$

**Table 2:** Skin friction versus embedded parameters using both cases.

$M$	$E$	$\phi_{1} ,\phi_{2}$	$$\begin{aligned} & f^{\alpha + 1} (0) \\ & {\text{TiO}}_{2} + {\text{Ag}},\alpha = 1, \\ \end{aligned}$$	$$\begin{aligned} & f^{\alpha + 1} (0) \\ & {\text{TiO}}_{2} ,\,\,\alpha = 1, \\ \end{aligned}$$	$$\begin{aligned} & f^{\alpha + 1} (0),\alpha = 0.90, \\ & {\text{TiO}}_{2} + {\text{Ag}}, \\ \end{aligned}$$	$$\begin{aligned} & f^{\alpha + 1} (0) \\ & {\text{TiO}}_{2} \,,\,\alpha = 0.90, \\ \end{aligned}$$
0.1	0.1	0.01	0.3536421321	0.345267312	0.3131223536421	0.30132313
0.2			0.3646512438	0.356224131	0.3234564651243	0.312356224
0.3			0.3762431213	0.367134232	0.3324537624312	0.323217134
	0.2		0.36210864214	0.352109756	0.3221786210864	0.314212109
	0.3		0.35244312674	0.341203521	0.3189015244312	0.302341203
	0.4		0.34195320134	0.330254376	0.30231454195320	0.291432025
		0.02	0.34573421564	0.332134210	0.3021045734215	0.292134210
		0.03	0.34953210214	0.334132908	0.3032149532102	0.294132908
		0.04	0.35321075642	0.336321089	0.3102313210756	0.296321089

When $$\Pr = 21.$$

**Table 3:** Heat Transfer rate using both cases.

${\text{Ec}}$	$\phi_{1} ,\phi_{2}$	$$\begin{aligned} & \Theta^{\alpha } (0),\alpha = 1, \\ & {\text{TiO}}_{2} + {\text{Ag}}, \\ \end{aligned}$$	$$\begin{aligned} & \Theta^{\alpha } (0),\alpha = 1, \\ & {\text{TiO}}_{2} ,\, \\ \end{aligned}$$	$$\begin{aligned} & \Theta^{\alpha } (0),\alpha = 0.90, \\ & {\text{TiO}}_{2} + {\text{Ag}},\,\,\, \\ \end{aligned}$$	$$\begin{aligned} & \Theta^{\alpha } (0),\alpha = 0.90, \\ & {\text{TiO}}_{2} \,,\,\alpha = 0.90, \\ \end{aligned}$$
0.1	0.01	0.521321089	0.2567901235	0.3123211321	0.2321567901
0.2		0.6126702121	0.3425687101	0.3934561267	0.31254256871
0.3		0.7013452167	0.35130654321	0.6823470134	0.332151306543
	0.02	0.77232145621	0.3821076542	0.7121245721	0.35348210765
	0.03	0.8307632103	0.40134216432	0.7834202307	0.39801342164
	0.04	0.9520876321	0.44852764907	0.8312332151	0.410234852764

When $$\Pr = 21.$$

**Table 4:** Comparison with^8,9^ considering integer order and common parameters.

$\Pr$	Present $\Theta^{\prime}(0)$	Paullet and Weidman^8^ $\Theta^{\prime}(0)$	Hamad and Ferdows^9^ $\Theta^{\prime}(0)$
21	0.94736210	0.94747422	0.947453211
21.5	0.87542612	0.87553824	0.8755132226
22	0.72863213	0.72874426	0.7287231063

When $$\lambda = \frac{a}{b}$$.

## Conclusion

In the current study, we scrutinized the fractional behavior of the two-dimensional stagnation point flow of the hybrid nanofluid consisting of TiO_2_ and Ag nanoparticles across a stretching sheet. For the purposes of testing and medication, blood is designated as a base fluid. The fluid movement over a stretching layer is subjected to electric and magnetic fields. The modeled equations have been solved via fractional code FDE-12 based on the Caputo derivative. The following findings have been drawn:

- The titanium dioxide TiO_2_ and silver Ag is one of the most suitable nanocomposites for blood use, due to their ability to suppress bacterial growth and prevent the development of new cell structures.
- The drifting velocity generated by the electric field *E* significantly improves the velocity and heat transition rate of blood.
- The uses of titanium dioxide TiO_2_ and silver Ag nanoparticles in the base fluid are more efficacious for velocity and energy propagation.
- The magnetic effect retards the fluid velocity, while enhances the thermal energy profile $\Theta \left( \eta \right)$.
- The fluid has a maximum velocity at $\alpha = 1,$ but gradually the fluid velocity start declination with decreasing values of $\alpha .$
- The fractional model is more generalized and applicable than the classical one.

## List of symbols

$$u,v$$: Velocity component
$$\alpha$$: Fractional order
$$\lambda$$: Mixed convection
$$c_{p}$$: Specific heat
*E*: Electric field
$\phi_{1}$: Volume friction of titanium dioxide
$\phi_{2}$: Volume friction of silver
*M*: Magnetic field
$k_{hnf}$: Hybrid nanofluids thermal conductivity
Ec: Eckert number
Pr: Prandtl number
Re: Reynold number
$$\Theta$$: Dimensionless temperature
$u_{w}$: Stretching velocity of sheet
$u_{e} \left( x \right)$: *x*-component of velocity at the edges
$$\rho_{hnf}$$: Nanofluids density
$$\rho$$: Density
$\mu_{hnf}$: Hybrid nanofluid dynamic viscosity
$\nu_{hnf}$: Hybrid nanofluid kinematic viscosity
$$Cf_{x}$$: Drag force
$${\text{Nu}}_{x}$$: Nusselt number
$$n_{f}$$: Nanofluid
$$h_{nf}$$: Hybrid nanofluid
TiO_2_: Titanium dioxide
